# PES1 is a biomarker of head and neck squamous cell carcinoma and is associated with the tumor microenvironment

**DOI:** 10.1002/cam4.5948

**Published:** 2023-04-19

**Authors:** Yaodong He, Junwei Xiang, Yunshan Li, Wenkai Huang, Feihan Gu, Yuanyin Wang, Ran Chen

**Affiliations:** ^1^ College & Hospital of Stomatology Key Laboratory of Oral Diseases Research of Anhui Province, Anhui Medical University Hefei China

**Keywords:** head and neck squamous cell carcinoma, immune infiltration, PES1, prognosis, TCGA

## Abstract

**Background:**

As a nucleolar protein associated with ribosome biogenesis in multiple cancer types, PES1 has been reported to be overexpressed, promoting cancer cell proliferation and invasion. However, in head and neck squamous cell carcinoma (HNSCC), the role of PES1 on the prognosis and immune infiltration remains unknown.

**Methods:**

Multiple databases and qRT‐PCR evaluated the expression of PES1 in HNSCC. The prognostic potential of PES1 in HNSCC patients was analyzed by Cox regression and Kaplan–Meier curves. Then, we used LASSO regression and stepwise multivariate Cox regression to construct the PES1‐related risk assessment model. In addition, the association between PES1 and tumor immune microenvironment and drug sensitivity was explored by R packages. Finally, we used cell function assays to explore in HNSCC if PES1 influences tumor growth and metastasis.

**Results:**

PES1 was significantly up‐regulated in HNSCC and closely correlated with HPV status, tumor stage, clinical grade, and TP53 mutation status. Survival analysis suggested that PES1 is associated with worse survival outcomes, acting as an independent prognostic indicator for HNSCC. Our model also performed well in terms of prognosis prediction. Furthermore, tumor‐infiltrating immune cells and antitumor drug susceptibility were negatively related to PES1 expression. Functionally, as for HNSCC cell lines in vitro, the knockdown of PES1 could inhibit proliferation, migration, and invasion.

**Conclusion:**

We have demonstrated that PES1 may be a promoter of tumor growth. PES1 holds excellent promise as a novel biomarker to assess the prognosis of patients with HNSCC and may guide immunotherapy.

## Graphical abstract

Flow chart of the present study. The TCGA‐HNSCC cohort was used as the exploration set, and samples from GSE13601, GSE25009, GSE30784, GSE41613, and GSE65858 were used as the validation sets.

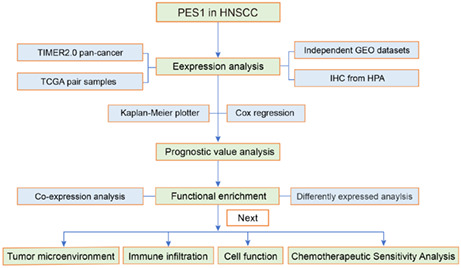

## 1 INTRODUCTION

As a common malignancy, Head and neck squamous cell carcinoma (HNSCC) can form in the mouth, pharynx, larynx, lip, nasal cavity, and salivary glands.^1^, ^2^ According to the reports, in 2021, more than 878,000 new cases of HNSCC will be diagnosed worldwide, with an estimated 444,000 deaths.^3^ Due to local recurrence and metastases in the cervical lymph nodes and the resistance to conventional chemotherapy, the rate of treatment failure and mortality in patients with advanced HNSCC is high. Effective multidisciplinary treatment for HNSCC remains limited, and the survival rate for patients with advanced HNSCC is only 34.9%.^4^, ^5^ Emerging research seeks predictive biomarkers from large cohorts of HNSCC patients to help clinicians diagnose accurately and predict clinical outcomes and inform patients with HNSCC the individualization of immunotherapy.^6^

Pescadillo ribosomal biogenesis factor 1 (PES1), also known as Pescadillo or NOP7, is located on chromosome 22q12.2 and contains 19 exons in humans and is a nucleolin protein that is essential for ribosome biogenesis, nucleoporin production, and cell growth.^7^, ^8^ Numerous studies have shown that PES1 is associated with tumor cell proliferation, invasion and metastasis in many types of cancers, including prostate,^9^ liver,^10^ pancreatic,^11^ thyroid,^12^ breast,^13^ ovarian,^14^ gastric^15^ and colon cancers.^16^ Some studies have also shown that PES1 is associated with the prognosis of some cancers.^10^, ^16^ Cheng et al.^17^ and Li et al.^14^ reported a novel function of PES1 that regulates the balance between ERα and ERβ protein levels. They found that PES1 enhanced the stability of ERα while targeting ERβ for proteasomal degradation, thereby increasing the protein levels of ERα and decreasing those of ERβ, which contributed to the development and progression of breast and ovarian cancers. Wang et al.^18^ reported that PES1 promotes tumorigenesis in hepatocellular carcinoma by regulating the PI3K/AKT pathway. It has been reported that PES1 interacts with BRD4 to enhance the expression of c‐Myc, thereby promoting cell growth and cellular resistance to extra‐terminal inhibitors in pancreatic cancer.^11^ In addition, PES1 activates Wnt/β‐Catenin signaling and stabilizes β‐Catenin in human cancer cells in a β‐Catenin‐dependent manner via siRNA‐mediated knockdown or CRISPR/Cas9 strategies.^19^ All these data suggest that PES1 plays an oncogenic role in various cancers. However, there are no relevant studies on the role of PES1 in the tumor microenvironment and prognosis of HNSCC.

By comprehensive bioinformatic analysis, this study explored the immune infiltration impact and prognostic value of PES1 in HNSCC. First, PES1 expression was compared between normal and carcinoma tissues and assessed the relationship of PES1 expression with the clinical feature of HNSCC patients. Subsequently, we evaluated the prognostic role of PES1 in HNSCC patients and established a PES1‐related risk model. Next, we comprehensively analyzed the immune infiltration landscape of HNSCC using multiple algorithms. Finally, we used cell function assays to explore if PES1 influences tumor growth and metastasis in HNSCC. We hope that PES1 can serve as clinical guidance for HNSCC patients.

## 2 MATERIALS AND METHODS

### 2.1 Data acquisition and gene expression analysis

We performed TIMER2 (http://timer.cistrome.org/) to PES1 expression in pan‐cancer.^20^ Then, we collected three datasets (accession: GSE25099, GSE30784, GSE13601) from GEO to explore the expression of PES1 in HNSCC patients. The immunohistochemical staining for PES1 expression was obtained by the Human Protein Atlas (HPA).^21^ Based on UALCAN (http://ualcan.path.uab.edu/analysis.html), we comprehensively analyzed PES1's expression in different clinical subgroups, including TP53 status, HPV status, nodal metastasis status, individual cancer stage, tumor grade, and gender, age, race.^22^

### 2.2 Prognostic value analysis

GEPIA2.0 (http://gepia2.cancer‐pku.cn/) and Kaplan–Meier plotter (https://kmplot.com/analysis/) plot the Kaplan–Meier survival curves of PES1.^23^, ^24^ The survival role of PES1 was further validated using clinical data from the GEO database (accession: GSE65858, GSE41613). We performed multivariate and univariate Cox regression analyses to identify whether the PES1 can be an independent prognostic predictor.

### 2.3 PES1‐related genes acquisition and functional enrichment analysis

Using LinkedOmics (http://www.linkedomics.org/), the HNSCC cohort from TCGA performed a Co‐expression analysis of PES1.^25^ For correlation analysis, genes with |cor| > 0.5 and adjusted *p*‐value <0.05 were considered co‐expressed genes by using the Pearson correlation test. Based on the median expression value of PES1, we divided the TCGA HNSCC samples into the high‐expression and low‐expression groups. We used the “limma” R package between two groups to identify differentially expressed genes (DEGs), with the filter |logFC| > 1.0 and *p*‐value <0.05. PES1‐related genes are the set of co‐expressed genes and differentially expressed genes.

### 2.4 Functional enrichment analysis of PES1 differential genes

Gene Set Enrichment Analysis (GSEA) and Gene Set Variation Analysis (GSVA) were used to explore PES1‐associated pathways and phenotypes and to compare biological functions between patients with high and low PES1. The Gene Ontology (GO) and Kyoto Encyclopedia of Genes and Genomes (KEGG) gene sets were obtained from the licensed portal. The subsets “c5.go.v2022.1.Hs.symbols.gmt” and “c2.cp.kegg.v7.4.symbols.gmt” were downloaded from the Molecular Signatures Database (MSigDB) to evaluate oncogenic or tumor‐associated pathways in HNSCC, and the limma, clusterProfiler, and GSVA packages in R were used for functional analysis.

### 2.5 Construction of risk assessment model

We conducted a univariate Cox regression analysis to obtain survival‐related PES1 genes. And then, we used stepwise multivariate Cox regression and Least Absolute Shrinkage and Selection Operator (LASSO) regression to establish a risk assessment model. Then, we calculated the Akaike information criterion (AIC) values for each attribute on the 5‐year receiver operating characteristic (ROC) curve to assess the cut‐off point high‐ and low‐risk scores. The “survival ROC,” “Survival,” and “Survminer” packages were utilized to evaluate the capability of the model's prediction.

### 2.6 Immune infiltration analysis

We used acknowledged methods to calculate the immune infiltration level of the patients with HNSCC, such as the CIBERSORT‐ABS, EPIC, MCPCOUNTER, QUANTISEQ, TIMER, XCELL, and then evaluated the relationship between the expression of PES1 and immune infiltration cell.^26^, ^27^, ^28^, ^29^, ^30^, ^31^ We also utilized the “ESTIMATE” R package to evaluate the tumor microenvironment (TME) status of each HNSCC sample, and the results were presented as immune/stromal/ESTIMATE scores.^32^ We used the “ggpubr” R package between the high‐ and low‐expression groups to visualize the differences in these scores. Moreover, we used the single‐sample Gene Set Enrichment Analysis (ssGSEA) to examine these groups' immune function differences. We further evaluated different immune pathways enriched in PES1 high‐ and low‐expression samples in the light of the immune‐related gene set c7.immunesigdb_HALLMARK using the “GSVA” R package.

To predict the immune response of PES1 to immunotherapy, the TIDE database was used to calculate TIDE scores and immune response rates for high‐PES1 and low‐PES1 subgroups, classified by their median expression. Patients with a higher TIDE score will have a higher chance of immune rejection, indicating a lower likelihood of benefiting from immunotherapy.^33^ Imvigor210 data were also downloaded for predicting immunotherapy response.^34^ Finally, we used the TIDE database to assess the predictive ability of PES1 and other markers for the HNSCC immunotherapy cohort, with higher AUCs associated with better marker performance.

### 2.7 Analysis of chemotherapeutic sensitivity

To evaluate the impact of PES1 in the clinic for HNSCC treatment, the half‐maximal inhibitory concentration (IC50) of chemotherapeutic drugs in patients with HNSCC was figured by the “pRRophetic” R package based on anticancer drug sensitivity information from the GDSC project.^35^, ^36^ Finally, we used the Wilcoxon rank‐sum test to examine the differences between the high‐ and low‐expression groups in the chemotherapeutic sensitivity. The findings were shown in box plots. Furthermore, we used the NCI‐60 cancer cell line from CellMiner to analyze the connection between PES1 expression and drug response.^37^

### 2.8 Patient tissue samples and cell lines

Twenty‐seven pairs of quickly frozen HNSCC and surrounding healthy tissue were obtained from patients through the operation. Before the procedure, all experimental patient samples received no other therapies, and a thorough pathologic analysis validated all HNSCC tissues. Each patient signed a written informed permission form, and the Medical Ethics Committee approved the study at Anhui Medical University's Affiliated Stomatological Hospital. Each method was conducted as per appropriate regulations and guidelines.

HNSCC cell lines SCC4, SCC9, CAL27, HN4, HN6, and human normal oral epithelial keratinocytes (HOK) were purchased from Ninth People's Hospital Affiliated with Shanghai Jiaotong University School of Medicine. These cell lines were subjected to STR profiling and tested for mycoplasma contamination every 3 months. These cell lines were saved in DMEM (BI, Israel) supplemented with 10% fetal bovine serum (BI, Israel), 1% penicillin, and streptomycin (NCM). Moreover, in a humidified incubator containing 5% CO_2_, all these cell lines were cultured at 37°C.

### 2.9 RNA extraction and quantitative real‐time polymerase reaction (qRT‐PCR)

We used Thermo Fisher Scientific's TRIzol reagent (USA) to extract total RNA from tissues/cell lines. And then, we used a NanoDrop Spectrophotometer (Thermo Fisher Scientific, USA) to determine the concentration and purity of RNA. We used the Prime Script RT Master Mix from Takara (Cat. #RR047A) to reverse transcribe the RNA(total). Moreover, we used CFX96 Touch Real‐Time PCR Detection System (Bio‐Rad) per the manufacturer's protocol to conduct qRT‐PCR. For internal reference, GAPDH was used, and the reaction was conducted in two steps. The conditions for the reaction were: pre‐denaturation at 95°C for 30 s, denaturation at 95°C for 5 s, and annealing/extension at 60°C for 30 s for 50 cycles. The threshold cycle (Ct) approach was used to estimate expression, and the 2–ΔΔCt method calculated relative expression levels. Information about primers is shown in Table S1.

### 2.10 Western blot

In RIPA buffer (Epizyme), Cell samples were harvested and then centrifuged at 12,000 × g and 4°C for 15 min. We collected supernatants and used a BCA Kit (Beyotime) to calculate protein concentrations. And 40–60 μg of each sample was taken, and the SDS‐PAGE electrophoresis separated proteins and transferred them into polyvinylidene fluoride membranes. The membranes were incubated with primary antibodies against PES1 (Proteintech, #13553‐1‐AP) and GAPDH (Proteintech, #60004‐1‐Ig) overnight at 4°C after 2 h, closed with 5% milk powder at room temperature. At room temperature, the signal was incubated with a secondary antibody for 1 h after washing 3 times with TBST. Then, according to the manufacturer's recommendations, the signal was detected by enhanced chemiluminescence.

### 2.11 Cell transfection

We employed a negative control (NC) oligonucleotide and short interfering RNA (siRNA) to target PES1 (Table S2). For cell culture, SCC9 and CAL27 cells were seeded in 6‐well plates. Then, using Liposome 2000 (Invitrogen) in conformity to the manufacturer's instructions, at a final concentration of 50 nM, siRNAs or controls (General) were added to the cells.

### 2.12 Cell counting kit‐8 proliferation assay

In each well of a 96‐well plate, a total of 3000 cells were implanted. After transfection, 10 μL of CCK‐8 reagent was added to the culture medium at 0, 24, 48, and 72 h. Then, cells were incubated at 37°C for 1 h, and with a microplate reader, the absorbance was measured at 450 nm.

### 2.13 Colony formation assay

At a density of 800 cells per well, transfected SCC9 and CAL27 cells were inoculated in 24‐well plates and then incubated at 37°C in 5% CO_2_ for 2 weeks. Phosphate buffer solution (PBS), fixed with 4% paraformaldehyde, was used to wash cells for 30 min and stained with 0.1% crystal violet solution for 10 min. Then we counted and analyzed the colonies.

### 2.14 Wound healing assay

Eighty percent fusion was achieved after transfected SCC9 and CAL27 cells were injected onto a 12‐well plate. With the tip of a 10 μL pipette, single‐cell layers were scraped. After three PBS washes to remove cell debris, fresh media containing serum was added. At 0 and 24 h after scratching, three high‐magnification fields were taken to obtain typical images of cell migration. Using ImageJ, the scratch width was estimated.

### 2.15 Transwell migration and invasion assays

In the migration assay for the Transwell system(BD Biosciences), the upper chamber inoculated the transfected SCC9 and CAL27 cells, and 500 μL of medium containing 10% FBS was filled in the lower chamber. And 24 h later, we used a cotton swab to wipe off the cells remaining on the surface of the filter membrane, and the cells passing through the membrane were fixed with methanol and then stained with crystal violet solution. Three randomly selected fields of view (including the center and periphery of the membrane) counted the number of cells under an inverted microscope. In the invasion assay, Matrigel (BD Biosciences) was used to coat the filters of the Transwell system, and the other steps were similar to the migration assay.

### 2.16 Statistical analysis

The Wilcoxon rank‐sum test analyzed the expression level of PES1 between non‐tumor tissues and HNSCC tumor tissues downloaded from the GEO database. Multivariate and univariate analyses were employed for Cox regression. In addition, the Wilcoxon test analyzed TME scores, immune checkpoint expression, and IC50 between high‐ and low‐expression groups. Moreover, we estimated the correlation coefficient of PES1 expression and immune infiltration cell score by Pearson correlation analysis. All the above statistics used R version 4.1.1 software to perform. Unless otherwise stated, *p* < 0.05 was regarded as statistically significant.

## 3 RESULTS

### 3.1 High expression of PES1 in HNSCC

**FIGURE 1:**
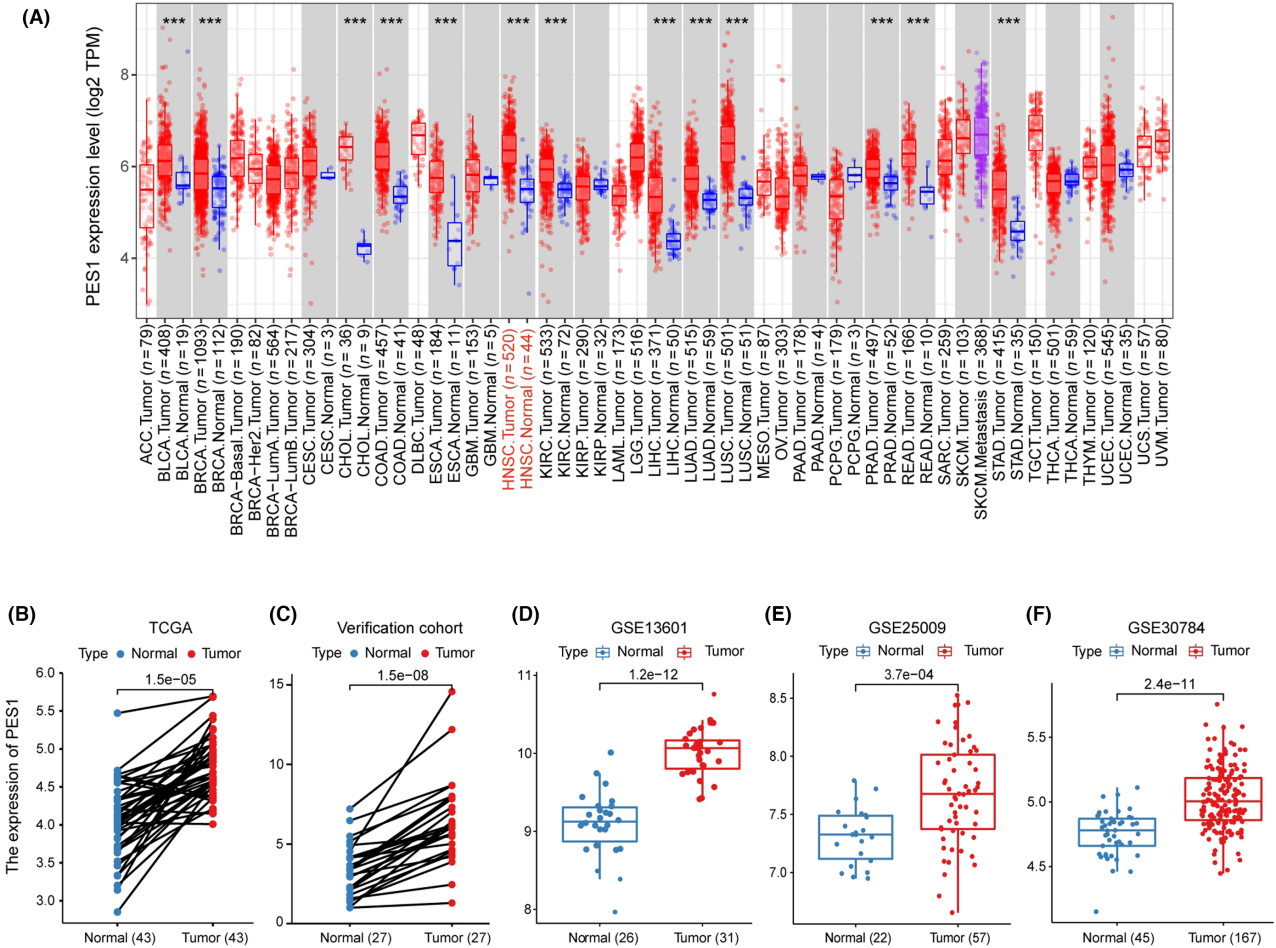
The expression profile of PES1 in HNSCC. (A) High or low expression of PES1 in various cancer tissues compared with normal tissues from the TCGA database. (B) The expression level of PES1 was higher in HNSCC tissue than in the adjacent normal tissue in the TCGA database. (C) The relative expression of PES1 in HNSCC tissues and adjacent normal tissues was detected by qRT‐PCR (*n* = 27). (D–F) The expression level of PES1 was more elevated in tumor tissues in GSE13601, GSE25099, and GSE30784 datasets.

To assess the differential PES1 expression in normal tissues and tumors, we analyzed the mRNA expression levels of pan‐cancer using the TIMER2 database. The results proved that the PES1 mRNA expression was upregulated in various cancer tissues than in corresponding normal tissues, including HNSCC. However, PES1 expression was decreased in Kidney Chromophobe (KICH) (Figure 1A). Notably, in HNSCC of HPV‐, the expression of PES1 was higher. Furthermore, PES1 expression was upregulated in 43 HNSCC samples compared to paired adjacent samples (Figure 1B). To further verify whether PES1 expression levels were upregulated in HNSCC tissues, we detected the upregulation of PES1 in 27 HNSCC tissues compared with adjacent normal tissues by qRT‐PCR, which was consistent with TCGA datasets (Figure 1C). Independent samples from GEO (accession: GSE13601, GSE25099, GSE30784) verified that PES1 was overexpressed in HNSCC (Figure 1D–F).

**FIGURE 2:**
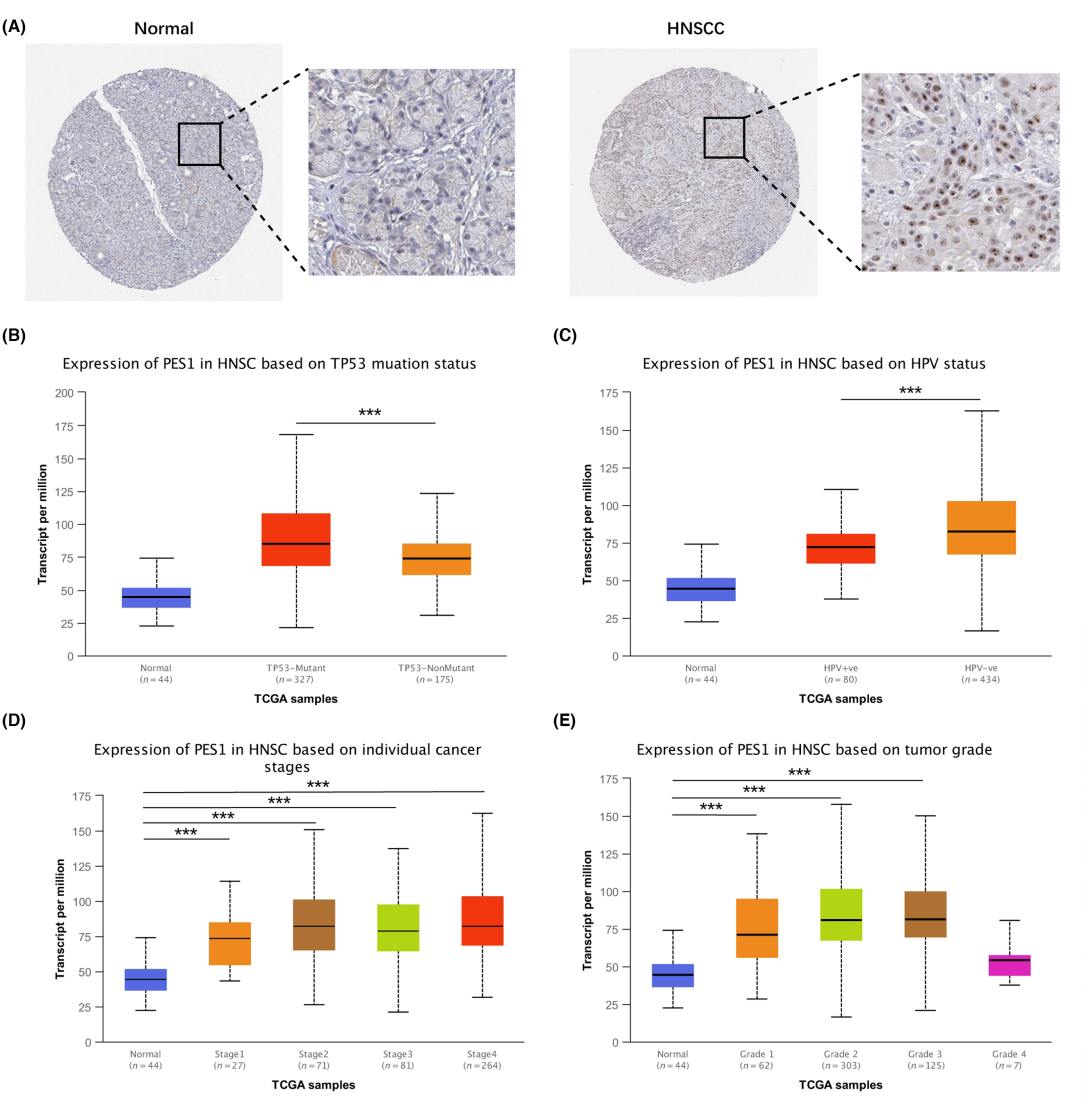
(A) Immunohistochemical staining of PES1 in normal oral tissues and HNSCC tissues from HPA. Comparison of PES1 expression in different subgroups of TP53 status (B), HPV status (C), tumor stage (D), and tumor grade (E) (*p < 0.05, **p < 0.01, ***p < 0.001).

As shown in Figure 2A, in normal salivary gland tissue, we observed low protein expression of PES1, while high protein expression was detected in HNSCC tissues. Finally, we used UNCLAN to assess the expression of PES1 between clinical subgroups of HNSCC and normal samples. As shown in Figure 2B–E, PES1 has significantly up‐regulated in the HPV‐negative group and TP53 mutant group. Furthermore, PES1 expression increased in tumor stage and pathological grade groups among the HNSCC cases. However, clinical subgroups showed no discernible changes.

### 3.2 Prognostic value of PES1 in HNSCC

**FIGURE 3:**
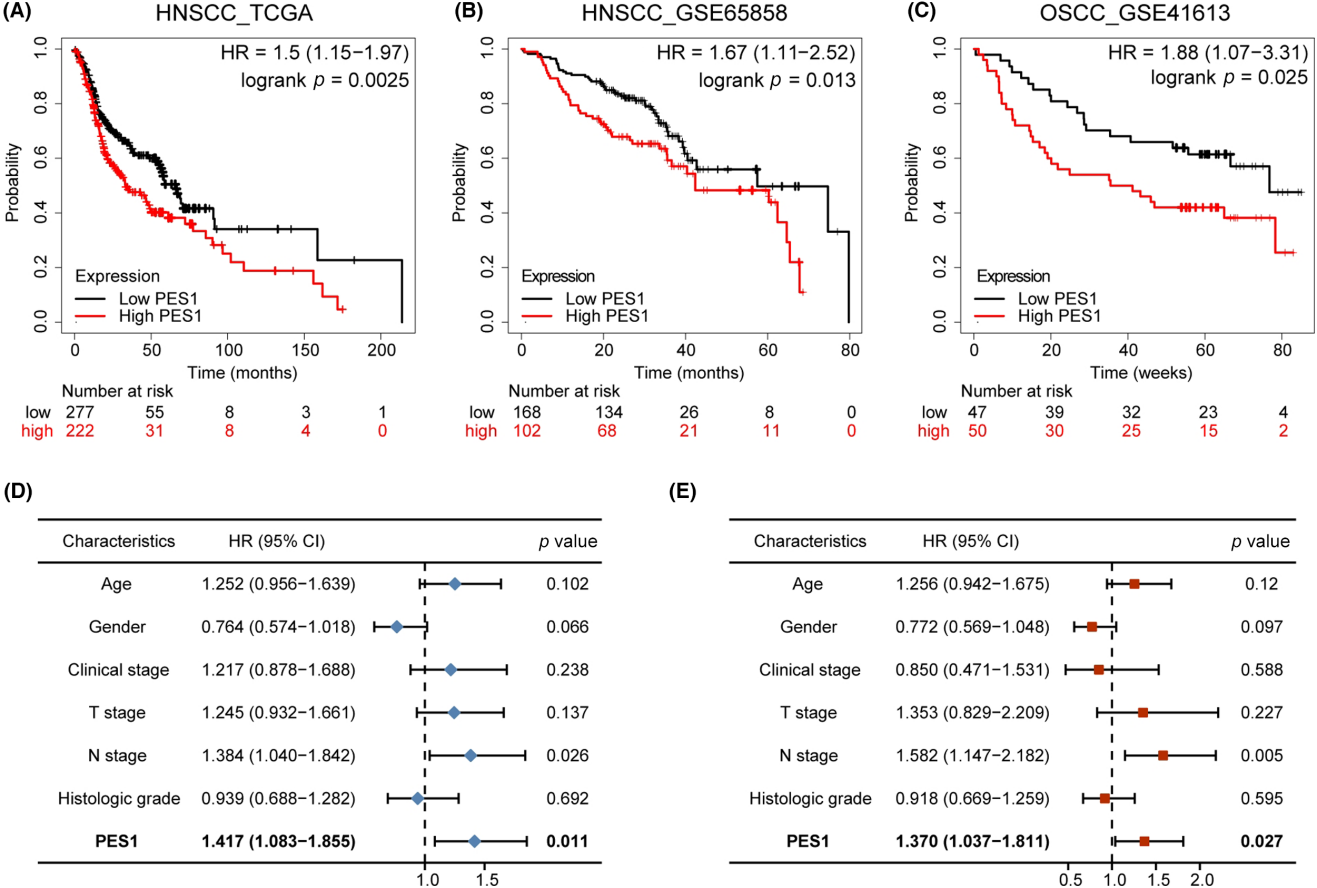
High‐PES1 expression was associated with a favorable prognosis in HNSCC samples. (A–C) Overall survival curves of TCGA and GEO patients by Kaplan–Meier plotter. (D, E) Univariate and multivariate analyses of overall survival and clinicopathologic characteristics in TCGA patients.

The Kaplan–Meier plotter investigated the influence of PES1 on overall survival in HNSCC. The survival curve proved that the expression of PES1 was obviously linked to a worse prognosis for HNSCC patients (HR = 1.5, *p* = 0.0025) (Figure 3A). Clinical data from the GEO database (accession: GSE65858, GSE41613) further confirmed the above results (Figure 3B,C). Subsequently, according to univariate Cox regression analysis, overall survival was related to PES1 and N stage, and multivariate regression analysis further indicated that in patients with HNSCC, PES1 could be a positive independent prognostic factor (HR = 1.370, *p* = 0.027) (Figure 3D,E; Table S3).

### 3.3 Enrichment analysis

**FIGURE 4:**
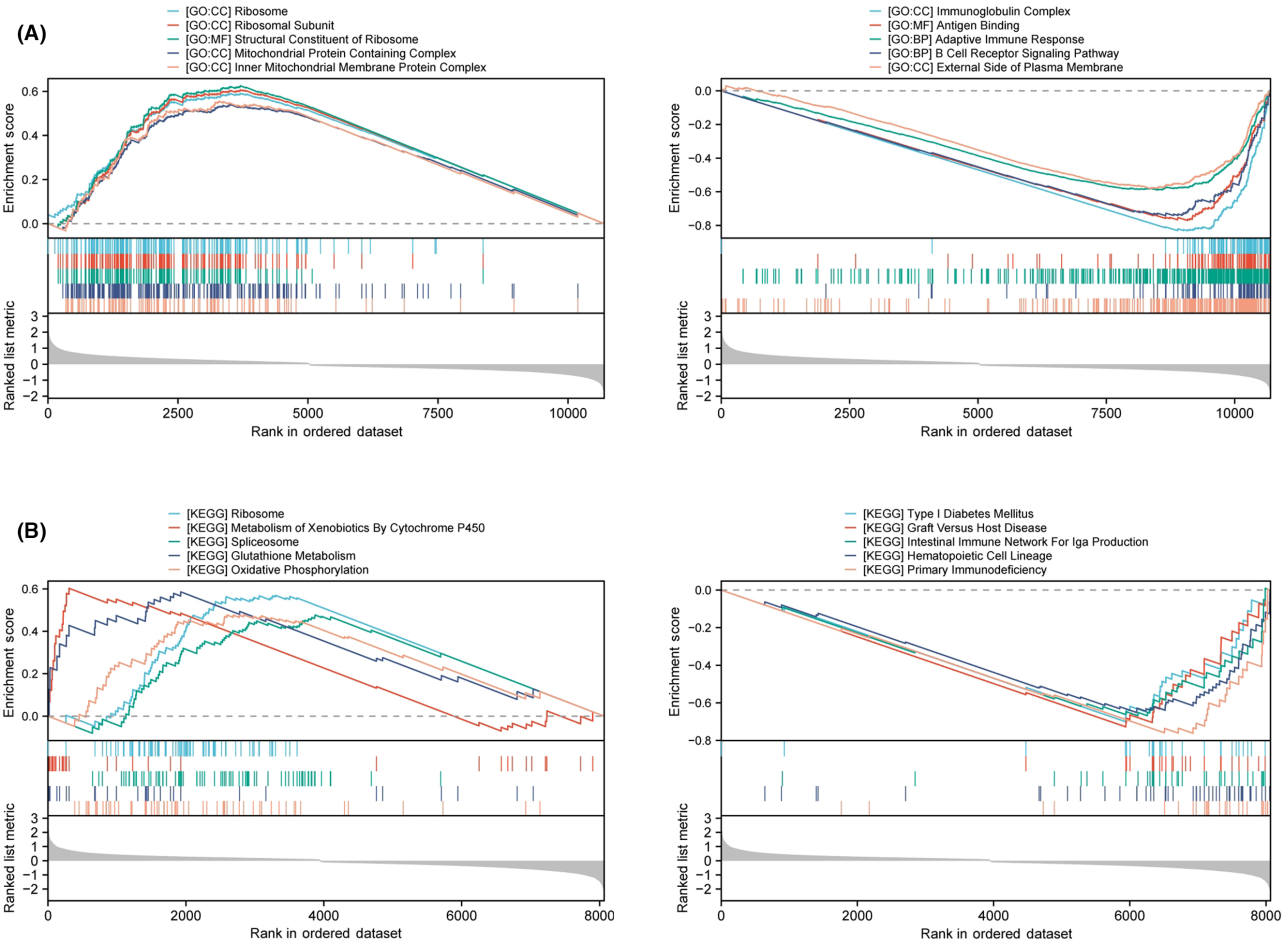
Functional enrichment analysis in HNSCC. (A, B) GSEA shows the top five GO terms and KEGG items enriched in the high and low PES1 expression groups.

To further investigate the molecular biological function of PES1 in HNSCC, we divided TCGA HNSCC samples into two groups according to PES1 levels and performed differential expression analysis. Based on the results of differential analysis, GSEA analysis was performed. We selected the highest enrichment pathway based on their normalized enrichment scores (NES). The results showed that PES1 differential genes were most significantly enriched in many immune response‐related processes, such as immunoglobulin complexes, antigen binding, adaptive immune responses, B‐cell receptor signaling pathways, primary immunodeficiency, intestinal immune network promoting IgA production, and hematopoietic stem cells (Figure 4A,B). GSVA is a method to estimate changes in genomic enrichment by converting gene expression matrices into genomic scoring matrices GSVA is a method for assessing changes in genomic enrichment by converting gene expression matrices into genomic scoring matrices. Combining GSVA and differential analysis can achieve differential analysis at the pathway level. We found that the T cell receptor signaling pathway, B cell receptor signaling pathway, JAK–STAT signaling pathway, Toll‐like receptor signaling pathway, and chemokine signaling pathway were suppressed in HNSCC samples with lower PES1 (Figure S1B). In addition, we noticed that ribosomal and mitochondrial‐related pathways were activated in HNSCC tissues with higher PES1, suggesting that PES1 may promote HNSCC by affecting cell proliferation and glycolysis (Figures 4A and S1A). In conclusion, the above results indicate that PES1 is essential in regulating the immune response in HNSCC.

### 3.4 Establishment and evaluation of PES1‐related risk model

**FIGURE 5:**
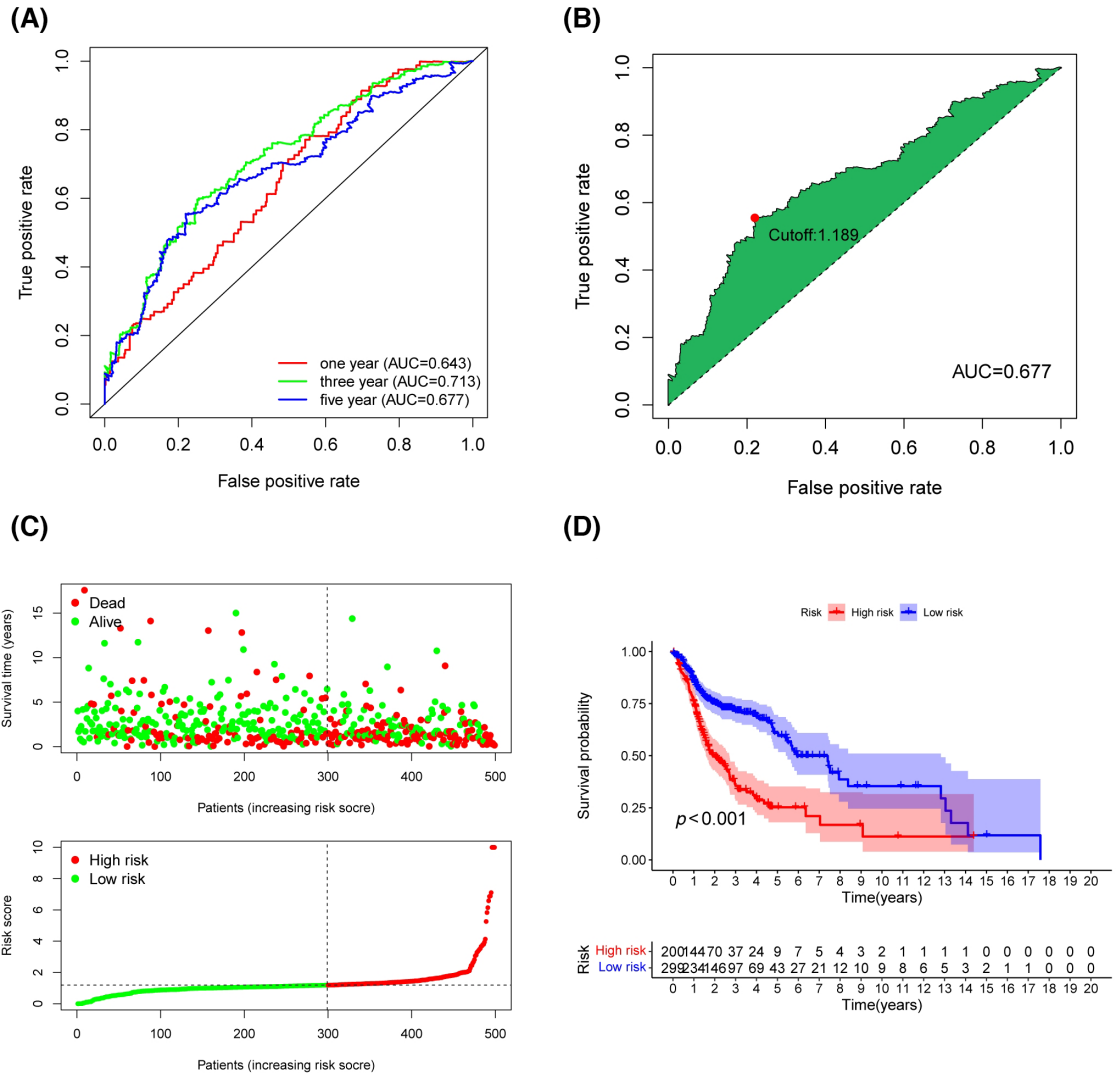
Risk Assessment Model for Prognosis Prediction (A) The risk model's 1‐, 3‐, and 5‐year ROC curves with AUC values. (B) Youden Index identifies the cut‐off risk score. (C) The risk score curve and the distribution of survival status of HNSCC patients. (D) Patients in the low‐risk group experienced a longer survival time tested by the Kaplan–Meier test.

LinkedOmics was utilized to explore the co‐expression profiles of PES1 in HNSCC, and a total of 106 co‐expressed genes were obtained (|cor| > 0.5, p < 0.05) (Figure S2A). Then, from the high‐ and low‐expression groups, we received 397 differentially expressed genes (Figure S2B). We determined 341 survival‐related PES1 genes (Table S4). Moreover, the prognostic variables were screened by LASSO regression analysis, and 26 PES1‐related genes were obtained (Figure S3A,B). Finally, the HNSCC risk assessment model was established by 12 prognosis‐related genes (Figure S3C,D). The predicted 1‐, 3‐, and 5‐year survival curves had areas under them of 0.643, 0.713, and 0.677, respectively, demonstrating that the risk assessment model is sensitive enough to predict survival. (Figure 5A). Furthermore, we divided patients into high‐risk and low‐risk groups and chose 1.189 as the cut‐off point (Figure 5B). As shown in Figure 5C,D, more deaths and a worse prognosis could be observed in the high‐risk group.

### 3.5 Relationship between PES1 and tumor microenvironment

**FIGURE 6:**
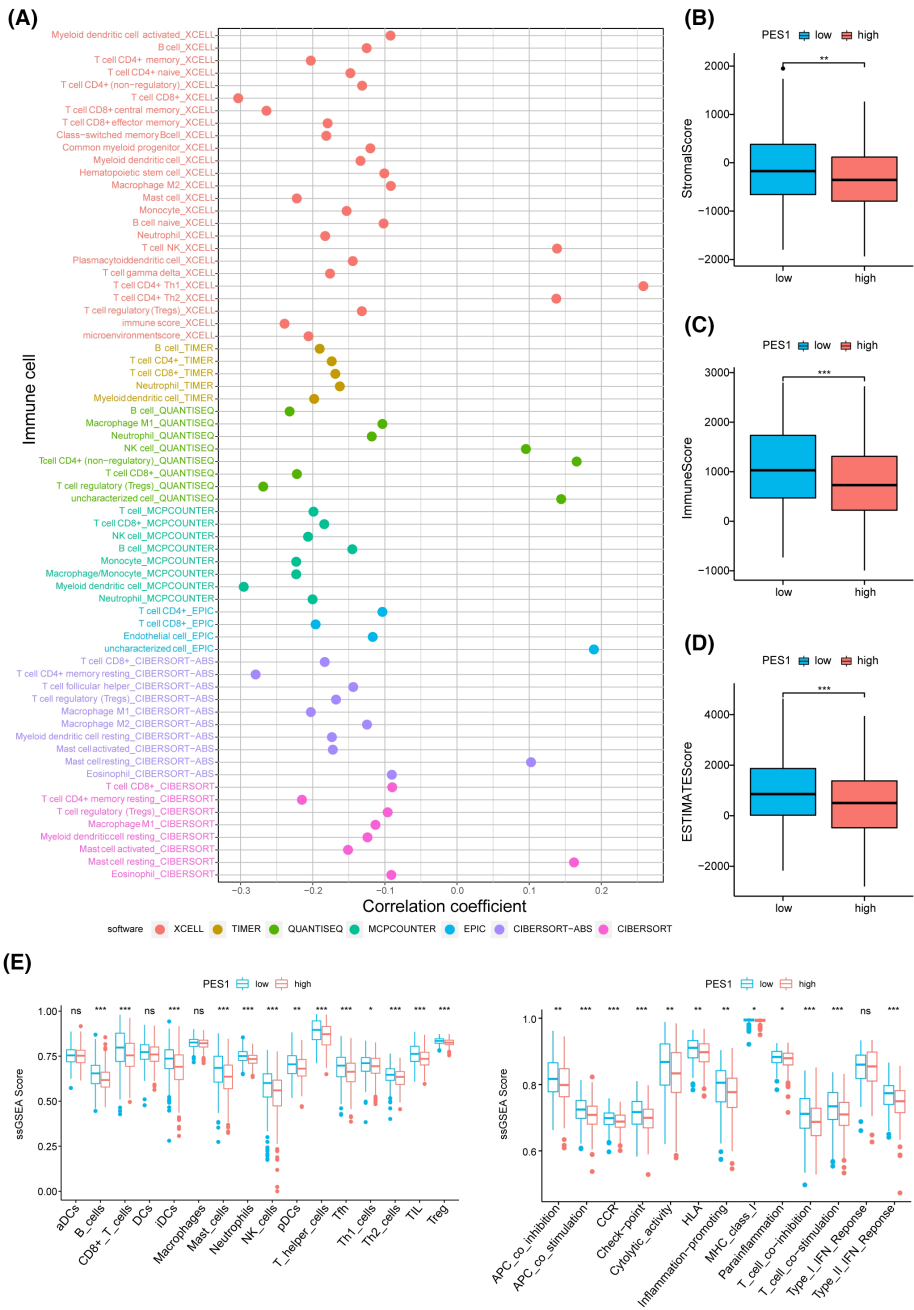
Estimation of immune‐infiltrating cells. (A) The PES1 expression was significantly negatively correlated with most immune cells. (B–E) The low‐PES1 group has higher TME scores and more active immune function (*p < 0.05, **p < 0.01, ***p < 0.001).

We calculated immune cell infiltration in HNSCC patients to further understand the impact of PES1 on the tumor microenvironment. As can be seen from the results, PES1 expression was negatively related to most immune cells, such as myeloid dendritic cells, CD4^+^ and CD8^+^ T cells, B cells, and macrophage cells (Figure 6A; Table S5). In addition, compared to the high expression group, the group with low expression of PES1 was higher in the ESTIMATE, immune and stromal scores (Figure 6B–D). The ssGSEA further suggested that low PES1 expression patients may have a more active immune response (Figure 6E).

### 3.6 The involvement of PES1 in response to immunotherapies

**FIGURE 7:**
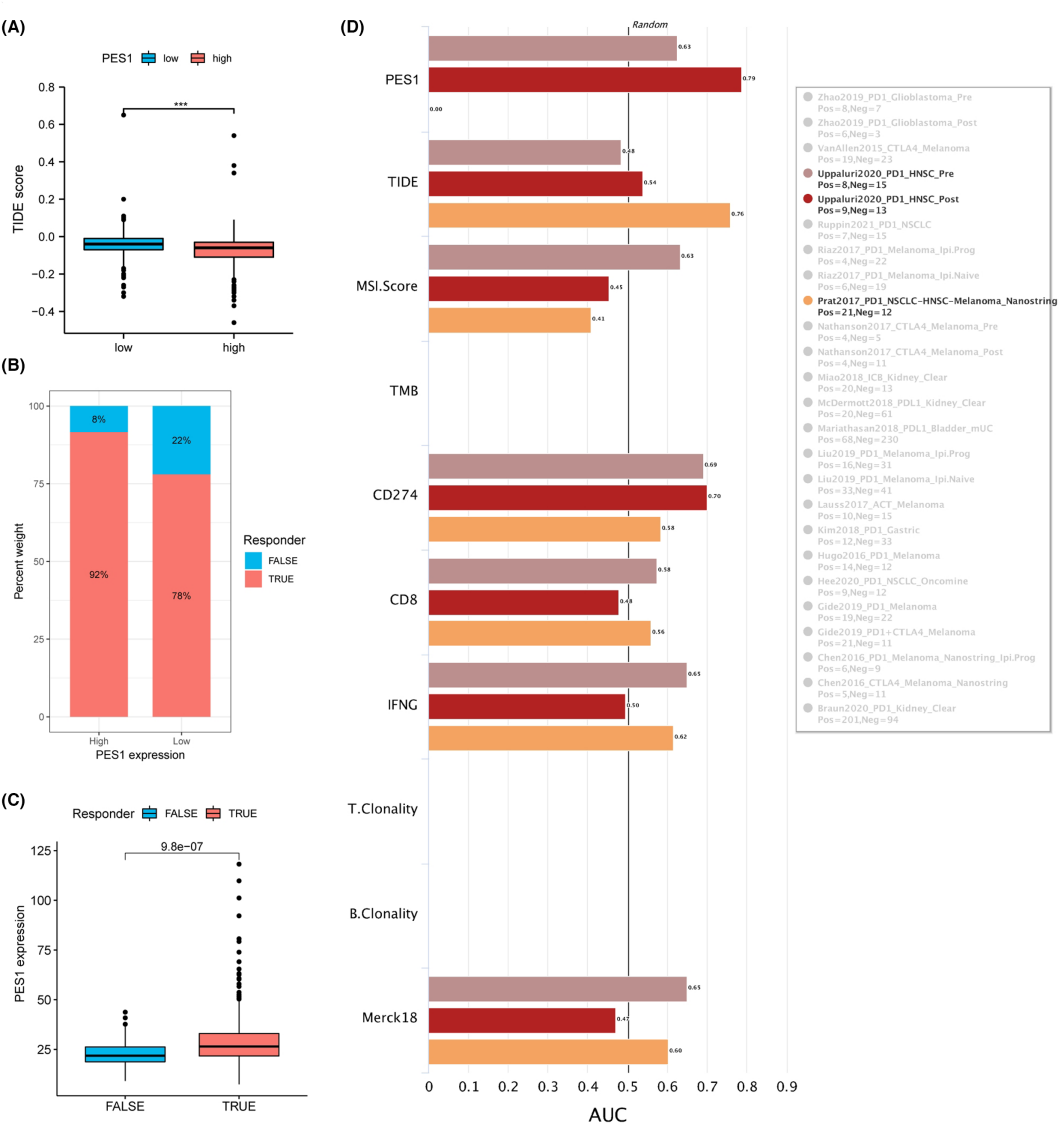
Immune responses of PES1 gene to immunotherapies. (A) Different distributions of TIDE scores between high and low PES1 expression. (B) The prediction of response rates of immunotherapies in patients with high and low PES1 expression. (C) Prediction of immune response by IMvigor210 cohort. (D) TIDE biomarker evaluation of PES1 in response to immunotherapy in HNSCC.

Based on previous publications and the above results, PES1 is closely associated with immunity. To further predict the immune response of PES1 to immunotherapy, the TIDE database and the IMvigor210 cohort were used, respectively. Notably, patients with high PES1 expression had lower TIDE scores and significantly higher response rates (Figure 7A,B). In addition, patients who responded clinically to immunotherapy also demonstrated higher PES1, indicating that patients with higher PES1 are more likely to benefit from immunotherapy (Figure 7C). The accuracy of PES1 in predicting response to immunotherapy was assessed using the area under the curve AUC of the ROC curve and compared with existing immunotherapy biomarkers, including TMB, TIDE, MSI score, Merck18, IFGN, CD8, and CD274. In the HNSCC cohort after PD‐1 treatment, the predictive performance of PES1 was higher than CD274, and both biomarkers had AUC values >0.7, predicting a positive response to immunotherapy with a high probability. Second, the predictive performance of PES1 was also comparable to other biomarkers in the HNSCC cohort prior to PD‐1 treatment (Figure 7D). The above results suggest that PES1 is a convincing indicator of immunotherapy for HNSCC.

### 3.7 PES1 promotes HNSCC cells proliferation, migration, and invasion in vitro

**FIGURE 8:**
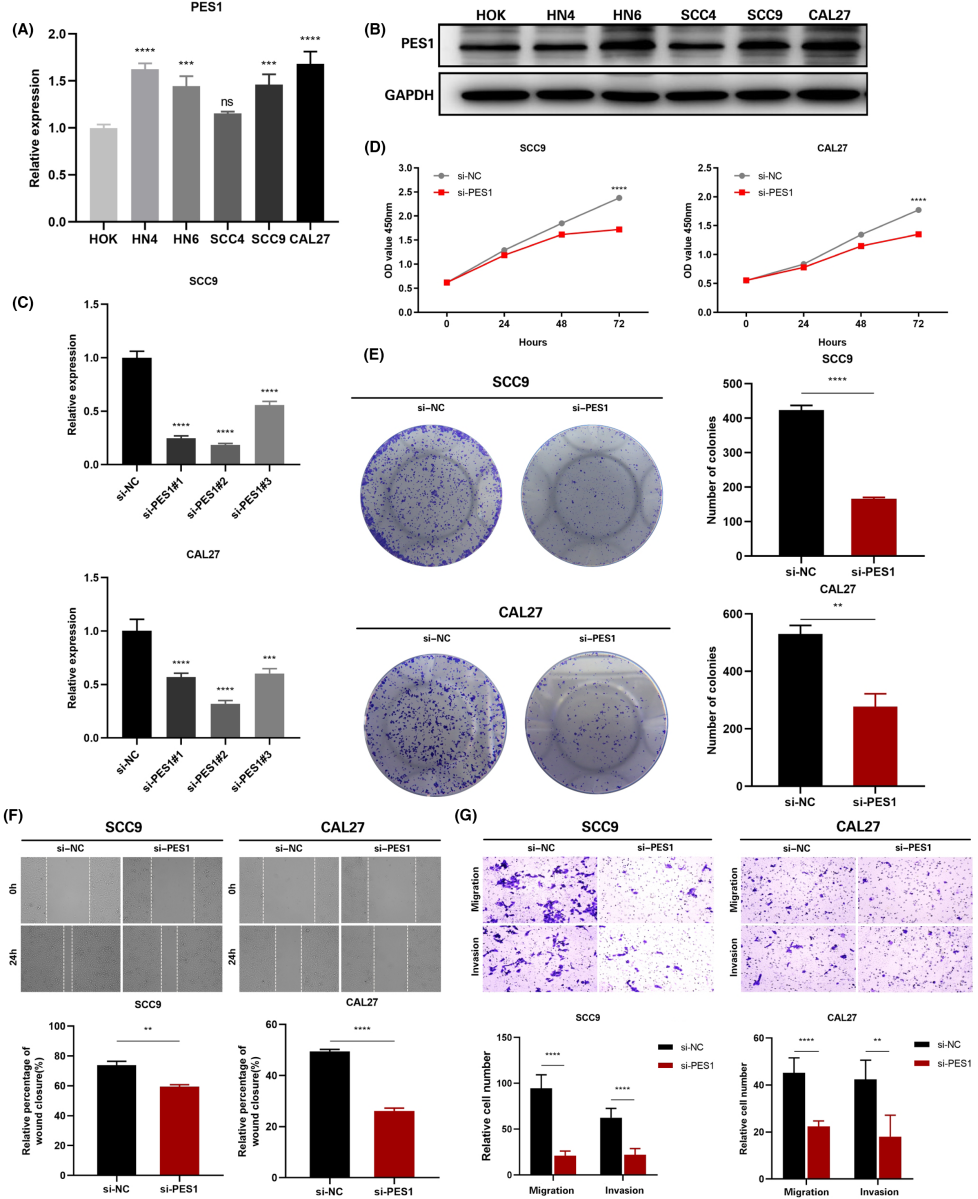
PES1 promotes the proliferation, migration, and invasion of HNSCC cells in vitro. (A) The relative expression of PES1 in the cells was detected by qRT‐PCR. (B) PES1 protein expression was detected by western blot in the cells. (C) qRT‐PCR analysis of PES1 expression in SCC9 and CAL27 cells treated with siRNAs. (D, E) The proliferation ability of SCC9 and CAL27 cells transfected with NC or si‐PES1 was examined by CCK‐8 and colony formation assay. (F, G) The migration and invasion ability of the cells was detected by wound healing and transwell assays. Data were showed as mean ± SD, *p < 0.05, **p < 0.01, ***p < 0.001, ****p < 0.0001.

In HOK and five HNSCC cell lines, the expression of PES1 was identified by qRT‐PCR, and the results revealed that five HNSCC cells expressed PES1 at a higher level than HOK cells (Figure 8A). Similarly, compared to HOK cells, in the five HNSCC cell lines, the PES1 protein expression was higher (Figure 8B). Ultimately, the SCC9 and CAL27 cell lines were selected by us to represent the knockdown cells of PES1. In SCC9 and CAL27 cells treated with siRNA fragments, according to qRT‐PCR data, PES1 expression was dramatically down‐regulated. The si‐PES1‐2 was chosen for further investigation among the three siRNAs because it showed the best silencing effectiveness in SCC9 and CAL27 cells (Figure 8C). The downregulation of PES1 inhibited the proliferative activity of HNSCC cells according to the CCK‐8 assay (Figure 8D). Colony formation analysis further showed that PES1 downregulation significantly inhibited the cell cloning ability of SCC9 and CAL27 (Figure 8E). Then, the influences of PES1 on HNSCC cell invasion and migration were examined by wound healing and transwell assays. The results showed that downregulation of the PES1 gene inhibited SCC9 and CAL27 cells invasion and migration ability (Figure 8F,G). These experiments proved that in HNSCC cells, PES1 could promote proliferation, migration, and invasion.

## 4 DISCUSSION

As a squamous cell tumor, HNSCC may occur anywhere in the head and neck.^38^ Although cancer treatment continues to advance, the overall survival rate for HNSCC patients, especially at advanced stages, remains undesirable because there are no excellent, trustworthy prognostic biomarkers.^39^ As a protein‐coding gene, PES1 is implicated in ribosome biogenesis and DNA replication.^8^, ^40^ PES1 has been discovered to be crucial for cell growth and may contribute to the development of tumors and oncogenic transformation.^41^ Li et al.^14^ showed that a decrease in PES1 caused a delay in the cell cycle's G2 phase and slowed ovarian cancer cell proliferation. Li et al.^15^ found that in vitro and in vivo, PES1 enhanced the growth of gastric cancer cells. Several studies have also shown that PES1 can be a prognostic biomarker for colon and liver cancer.^10^, ^16^ The current investigation discovered elevated PES1 expression levels in the TCGA and GEO databases, which are supported by the outcomes seen in tissue samples. In addition, our results confirmed that in HNSCC cells, down‐regulation of PES1 inhibited migration and proliferation.

In the present study, we found that PES1 expression was closely associated with clinicopathological features of HNSCC, such as grade, stage, TP53 mutation and HPV status. Tp53 (p53) is one of the most common oncogenes in human cancers. The p53 protein exerts antitumor effects by repairing DNA damage, regulating metabolism, normalizing reactive oxygen species levels, regulating non‐coding RNA expression, and promoting autophagy or ferric reduction.^42^ It was found that impaired ribosome biogenesis and loss of nucleolar integrity trigger nucleolar stress, which classically stabilizes the tumor suppressor p53 and induces cell cycle arrest and apoptosis.^19^ Statistically significant levels of PES1 expression were observed between the HPV‐infected and non‐HPV‐infected groups. HPV infection is associated with most oropharyngeal cancers and is a risk factor for HNSCC.^43^ Amplification of the cell cycle gene E2F1, TRAF3 deletion and mutation of the oncogene PIK3CA may regulate HPV‐associated tumors.^44^ The results suggest that PES1 may integrate HPV genetic information into the host genome.

Despite the past decade's multidisciplinary treatment strategies having made tremendous achievements, the survival rate of HNSCC patients did not improve significantly, and the 5‐year survival rate remains lower than 50%.^45^, ^46^ To develop treatment strategies and improve patient survival, identifying biomarkers that predict patient prognosis proves essential.^47^ The TCGA, GEO, and validation cohorts suggested that in HNSCC, compared to the patients with low PES1 expression, high PES1 expression has worse overall survival. According to univariate and multivariate Cox analyses, higher PES1 expression independently predicted a worse prognosis for HNSCC patients. To optimize the clinical decision‐making process for HNSCC, this study screened for PES1‐related genes and constructed a prognostic model. To evaluate the validity and accuracy of this prediction model, we performed ROC curve analysis for 1, 3, and 5 years. The results showed that as the AUC values were above 0.60, the model effectively predicted the prognosis of HNSCC patients. Based on the optimal cut‐off risk score determined by the Youden index, HNSCC patients were split into low‐ and high‐risk subgroups. According to the K‐M analysis, the risk model could distinguish good and lousy survival in patients with HNSCC.

In addition, we found some genes co‐expressed with PES1. In the high PES1 expression group, CTCFL (CCCTC‐binding Factor Like), NR0B1 (nuclear receptor subfamily 0, group B, member 1), and GAGE12J (G antigen 12 J) were expressed at higher levels. In the low PES1 expression group, CTCF is a chromatin insulator and multifunctional transcription factor, and mutations in this gene are associated with invasive breast cancer, prostate cancer, HNSCC and nephroblastoma.^48^ It was found that CTCF is involved in the transcriptional regulation of HOXA9 (homeobox A9), and the knockdown of CTCF inhibits proliferation, migration, invasion and drug resistance of HNSCC cells.^49^ KRT76 is the most significantly downregulated gene encoding a structural protein in human oral squamous cell carcinoma (OSCC) and is strongly associated with poor prognosis.^50^ In patients, KRT76 was detected in 100% of normal gingival oral epithelium, 44% of oral preneoplastic lesions, and 35% of OSCC biopsies.^50^ Sequeira et al.^51^ showed that knockout of KRT76 in mice resulted in enlarged spleens and lymph nodes, increased regulatory T cells, and elevated levels of pro‐inflammatory cytokines. Keratin KRT2 is a type II polypeptide predominantly located in the upper spinous and granular layers and expressed in the epidermis's late differentiation. In mild to moderate oral dysplasia with orthokeratosis, KRT2 is higher than in parakeratotic areas.^52^ Taken together, PES1 may be a predictive biomarker for the prognosis of HNSCC patients.

The GSEA results showed negative enrichment of KEGG pathways and GO terms for immune‐related pathways, including immunoglobulin complexes, antigen binding, adaptive immune responses, B‐cell receptor signaling pathways, and intestinal immune networks promoting IgA production, findings that prompted us to consider the role of PES1 in cancer immunology. Interestingly, pathways associated with tumor growth are also present in the PES1 high‐expression group. These pathways include structural components of ribosomes, mitochondrial protein complexes, oxidative phosphorylation, and glutathione metabolism. New evidence suggests that cancer cells harbor a specialized class of ribosomes (onco‐ribosomes) that facilitate oncogenic translation programs, regulate cellular function, and promote metabolic remodeling.^53^ Mutations in ribosomal proteins lead to ribosomal disease, associated with an increased risk of developing malignancies. Recent studies linking mutations in ribosomal proteins and abnormal ribosomes to poor prognosis have highlighted ribosome‐targeted therapy as a promising approach for the treatment of cancer patients.^54^ Mechanisms of acquired resistance to cisplatin have been reported in the literature due to enhanced drug detoxification systems due to high levels of scavengers such as glutathione/metallothionein, increased tolerance to damaged DNA, increased DNA repair mechanisms or metabolic rewiring of cells to escape cisplatin‐induced death.^55^, ^56^ The higher level of mitochondrial fusion processes in drug‐resistant cells compared to chemotherapy‐sensitive tumor cells suggests that mitochondrial fusion can promote cell survival through the efficient production of ATP and its transport.^57^ In addition to ATP production, oxidative phosphorylation is a primary source of ROS in mitochondria and throughout the cell. Several studies have shown that cisplatin‐induced cytotoxicity is closely associated with ROS production.^58^, ^59^ HNSCC samples were divided into high‐ and low‐risk groups based on the median level of PES1 expression. GSVA showed that among tumor immunological pathways, PES1 was negatively associated with multiple immune‐related pathways, such as NK cell‐mediated cytotoxicity, T‐cell receptors, B‐cell receptors, and Toll‐like receptor signaling pathways. These results imply that PES1 regulates several necessary functional oncogenic modules in tumors and that PES1 proteins or transcripts are involved in regulating the potential immunogenicity and immunotherapeutic effects of various cancers, thus promoting tumor patient progression and reducing prognosis. However, each pathway does not exist in an isolated state; they form a complex network. Therefore, the cross‐regulation of the various cascades in which PES1 is involved seems to provide opportunities for clinical treatment and subsequent studies.

Tumor immune infiltrating cells are an essential component of the tumor microenvironment and have been shown to play an important role in tumor proliferation and metastasis.^60^ An immunohistochemical study showed that tumors infiltrated by tumor‐infiltrating lymphocytes were associated with better outcomes in patients with HNSCC.^61^ In this study, we found that PES1 was negatively correlated with immune cells such as CD8^+^ T cells, macrophages, B cells, Treg cells, and neutrophils, and may be one of the relevant genes affecting the tumor microenvironment of oral squamous cell carcinoma. In addition, we found that ImmuneScore, StromalScore and ESTIMATEScore were lower in the high PES1 expression group, suggesting that the high PES1 expression group may be in an immunosuppressed state. CD8^+^ T lymphocyte dysfunction and failure were characterized by the upregulation of immunosuppressive molecules, such as PD‐1 and CTLA‐4, which suppressed the activation of CD8^+^ T lymphocytes. Persistent suppression in the tumor microenvironment may result in the inability of infiltrating CD8^+^ T lymphocytes to kill tumor cells, ultimately leading to tumor immune escape.^62^ The involvement of tumor‐associated macrophages (TAM) in HNSCC is an established fact: high TAM numbers in TME are associated with lymph node metastasis and advanced stages of HNSCC.^63^, ^64^ Studies have also shown a link between TAM and epithelial‐mesenchymal transition in OSCC.^65^ In HNSCC, aggregates of infiltrating B cells can be observed at the junction with normal tissue, in tertiary lymphoid structures, and occasionally within tumor nodes. Infiltration of B cells is effective in arresting tumor progression, and mechanisms for this arrest include promoting immunoglobulin production, stimulating T lymphocyte expression, and generating antibody‐dependent direct killing.^66^ Treg cells play an essential role in treating head and neck tumors as they effectively fight off cancer. Treg cells regulate lymphocyte activity and prevent tumor growth, thus acting as a disease prevention agent.^67^ Neutrophils account for about 60% of the total white blood cells. Therefore, they can act as a first barrier against inflammation and infection. Studies have shown that infiltration of multiple tumor cells may lead to advanced cancers and that the presence of these cells may affect the outcome of treatment of advanced cancers.^68^ These results suggest that the tumor microenvironment with high PES1 expression exhibits highly immunosuppressive properties, which provide the microenvironmental conditions for PES1 to promote tumor development, invasion, and metastasis.

Immunotherapy has revolutionized the treatment of cancer. Modern cancer immunotherapy has been developed over 50 years, and it was first applied to hematologic malignancies.^69^, ^70^ As for solid tumors, years of basic and clinical research have provided the rationale for investigating Immune Checkpoint Blockade (ICB), with treatments ranging from cellular therapies using tumor‐specific T cells, including cells expressing transgenic T Cell Receptors (TCR) and chimeric antigen receptors (CAR), to cells targeting crucial immune checkpoint molecules. CAR to antibodies that target important immune checkpoint molecules, such as PD‐1.^71^ Unfortunately, many patients do not respond to immunotherapy. Therefore, identifying new approaches to improve the efficacy of immunotherapeutic modalities is an area of active research. With the biomarker assessment module of the TIDE website, we evaluated the accuracy of PES1 in five ICB HNSCC cohorts and compared it with other published biomarkers associated with tumor immune evasion, including CD274 (PD‐L1), MSI Score, CD8, Merck18, and IFNG. We found that PES1 predicted a substantial likelihood of response to immunotherapy. Further analysis showed that patients with high PES1 expression responded well to ICB treatment. The rationale of the TIDE algorithm is to exploit two critical mechanisms of tumor immune evasion: inducing T cell dysfunction in tumors with high infiltration of cytotoxic T lymphocytes and blocking T cell infiltration in tumors with low levels of cytotoxic T lymphocytes, thereby capturing the patient's immunotherapeutic response.^72^ Therefore, we hypothesize that PES1 may play an integral role in forming a suppressive immune microenvironment and ultimately influence the immunotherapeutic response.

However, our work also has certain limitations. First, the prognostic value of PES1 must be validated in an actual clinical cohort. The database used in this study lacks post‐translational modifications and, therefore, cannot fully elucidate how these modifications affect the function of PES1. Furthermore, the upregulation of PES1 provides only indirect evidence of changes in TME, not direct evidence. The relationship between PES1 and TME is not well defined. Finally, the response of PES1 to immunotherapy requires further analysis of large‐scale protein sequencing or immunohistochemistry of PES1 to assess their relationship and validation by subsequent preclinical and functional studies. More work is needed to optimize clinical efficiency before it can be considered a practical therapeutic option.

## 5 CONCLUSION

In summary, our study shows that in HNSCC tissues, PES1 is highly expressed, and high expression of PES1 is related to decreased immune cell infiltration and survival in HNSCC. Moreover, we highlight the critical role of PES1 in the course of HNSCC and its potential prognostic and therapeutic value.

## AUTHOR CONTRIBUTIONS

**Yaodong He:** Writing – original draft (equal). **Junwei Xiang:** Writing – original draft (equal). **Yunshan Li:** Data curation (equal). **Wenkai Huang:** Visualization (equal). **Feihan Gu:** Investigation (equal). **Yuanyin Wang:** Supervision (equal); writing – review and editing (equal). **Ran Chen:** Supervision (equal); writing – review and editing (equal).

## FUNDING INFORMATION

This study was supported by Research Fund Project of Anhui Institute of Translational Medicine (2022zhyx‐C86) and Anhui Medical University Student Innovation and Entrepreneurship Training Program (S202210366083).

## CONFLICT OF INTEREST STATEMENT

The authors declare no competing interests.

## ETHICS STATEMENT

The collection of the clinical samples was performed under the supervision of Institutional Review Boards of The First Affiliated Hospital of Anhui Medical University (approval no. PJ2023‐02‐13). All patients provided written informed consent to provide their tumor tissues for further study, according to the Declaration of Helsinki.

## Supporting information


Figure S1.

Figure S2.

Figure S3.

Table S1.

Table S2.

Table S3.

Table S4.

Table S5.

## Data Availability

The datasets generated and/or analyzed during the current study are available in the The Cancer Genome Atlas (TCGA) repository, https://tcga‐data.nci.nih.gov/tcga/.

## References

[1] Pfister DG , Spencer S , Adelstein D , et al. Head and neck cancers, version 2.2020, NCCN clinical practice guidelines in oncology. J Natl Compr Canc Netw. 2020;18(7):873‐898. 32634781 10.6004/jnccn.2020.0031

[2] Chow L . Head and neck cancer. N Engl J Med. 2020;382(1):60‐72. 31893516 10.1056/NEJMra1715715

[3] Sung H , Ferlay J , Siegel RL , et al. Global cancer statistics 2020: GLOBOCAN estimates of incidence and mortality worldwide for 36 cancers in 185 countries. CA Cancer J Clin. 2021;71(3):209‐249. 33538338 10.3322/caac.21660

[4] Mcdermott JD , Bowles DW . Epidemiology of head and neck squamous cell carcinomas: impact on staging and prevention strategies. Curr Treat Options Oncol. 2019;20(5):43. 31011837 10.1007/s11864-019-0650-5

[5] Kitamura N , Sento S , Yoshizawa Y , Sasabe E , Kudo Y , Yamamoto T . Current trends and future prospects of molecular targeted therapy in head and neck squamous cell carcinoma. Int J Mol Sci. 2020;22(1):240. 33383632 10.3390/ijms22010240 PMC7795499

[6] Wang J , Chen X , Tian Y , et al. Six‐gene signature for predicting survival in patients with head and neck squamous cell carcinoma. Aging (Albany NY). 2020;12(1):767‐783. 31927533 10.18632/aging.102655 PMC6977678

[7] Nakaguro M , Kiyonari S , Kishida S , et al. Nucleolar protein PES1 is a marker of neuroblastoma outcome and is associated with neuroblastoma differentiation. Cancer Sci. 2015;106(3):237‐243. 25557119 10.1111/cas.12598 PMC4376431

[8] Du YC , Stillman B . Yph1p, an ORC‐interacting protein: potential links between cell proliferation control, DNA replication, and ribosome biogenesis. Cell. 2002;109(7):835‐848. 12110181 10.1016/s0092-8674(02)00773-0

[9] Jiang Z , Zhang Y , Chen X , et al. MicroRNA‐1271 impedes the development of prostate cancer by downregulating PES1 and upregulating ERbeta. J Transl Med. 2020;18(1):209. 32448371 10.1186/s12967-020-02349-1 PMC7245853

[10] Fu Z , Jiao Y , Li YQ , et al. PES1 in liver cancer: a prognostic biomarker with tumorigenic roles. Cancer Manag Res. 2019;11:9641‐9653. 31814761 10.2147/CMAR.S226471 PMC6861535

[11] Jin X , Fang R , Fan P , et al. PES1 promotes BET inhibitors resistance and cells proliferation through increasing c‐Myc expression in pancreatic cancer. J Exp Clin Cancer Res. 2019;38(1):463. 31718704 10.1186/s13046-019-1466-7 PMC6852745

[12] Qiu YB , Liao LY , Jiang R , et al. PES1 promotes the occurrence and development of papillary thyroid cancer by upregulating the ERalpha/ERbeta protein ratio. Sci Rep. 2019;9(1):1032. 30705367 10.1038/s41598-018-37648-7 PMC6355968

[13] Li S , Wang M , Qu X , et al. SUMOylation of PES1 upregulates its stability and function via inhibiting its ubiquitination. Oncotarget. 2016;7(31):50522‐50534. 27409667 10.18632/oncotarget.10494 PMC5226600

[14] Li J , Zhuang Q , Lan X , Zeng G , Jiang X , Huang Z . PES1 differentially regulates the expression of ERalpha and ERbeta in ovarian cancer. IUBMB Life. 2013;65(12):1017‐1025. 24376209 10.1002/iub.1228

[15] Li J , Zhou X , Lan X , Zeng G , Jiang X , Huang Z . Repression of PES1 expression inhibits growth of gastric cancer. Tumour Biol. 2016;37(3):3043‐3049. 26423399 10.1007/s13277-015-4069-8

[16] Xie W , Feng Q , Su Y , et al. Transcriptional regulation of PES1 expression by c‐Jun in colon cancer. Plos One. 2012;7(7):e42253. 22860098 10.1371/journal.pone.0042253 PMC3408486

[17] Cheng L , Li J , Han Y , et al. PES1 promotes breast cancer by differentially regulating ERalpha and ERbeta. J Clin Invest. 2012;122(8):2857‐2870. 22820289 10.1172/JCI62676 PMC3408741

[18] Wang J , Sun J , Zhang N , et al. PES1 enhances proliferation and tumorigenesis in hepatocellular carcinoma via the PI3K/AKT pathway. Life Sci. 2019;219:182‐189. 30630006 10.1016/j.lfs.2018.12.054

[19] Dannheisig DP , Bachle J , Tasic J , Keil M , Pfister AS . The Wnt/beta‐catenin pathway is activated as a novel nucleolar stress response. J Mol Biol. 2021;433(2):166719. 33221336 10.1016/j.jmb.2020.11.018

[20] Li T , Fu J , Zeng Z , et al. TIMER2.0 for analysis of tumor‐infiltrating immune cells. Nucleic Acids Res. 2020;48(W1):W509‐W514. 32442275 10.1093/nar/gkaa407 PMC7319575

[21] Uhlen M , Zhang C , Lee S , et al. A pathology atlas of the human cancer transcriptome. Science. 2017;357(6352):eaan2507. 28818916 10.1126/science.aan2507

[22] Chandrashekar DS , Bashel B , Balasubramanya S , et al. UALCAN: a portal for facilitating tumor subgroup gene expression and survival analyses. Neoplasia. 2017;19(8):649‐658. 28732212 10.1016/j.neo.2017.05.002 PMC5516091

[23] Lanczky A , Gyorffy B . Web‐based survival analysis tool tailored for medical research (KMplot): development and implementation. J Med Internet Res. 2021;23(7):e27633. 34309564 10.2196/27633 PMC8367126

[24] Tang Z , Kang B , Li C , Chen T , Zhang Z . GEPIA2: an enhanced web server for large‐scale expression profiling and interactive analysis. Nucleic Acids Res. 2019;47(W1):W556‐W560. 31114875 10.1093/nar/gkz430 PMC6602440

[25] Vasaikar SV , Straub P , Wang J , Zhang B . LinkedOmics: analyzing multi‐omics data within and across 32 cancer types. Nucleic Acids Res. 2018;46(D1):D956‐D963. 29136207 10.1093/nar/gkx1090 PMC5753188

[26] Becht E , Giraldo NA , Lacroix L , et al. Estimating the population abundance of tissue‐infiltrating immune and stromal cell populations using gene expression. Genome Biol. 2016;17(1):218. 27765066 10.1186/s13059-016-1070-5 PMC5073889

[27] Aran D , Hu Z , Butte AJ . XCell: digitally portraying the tissue cellular heterogeneity landscape. Genome Biol. 2017;18(1):220. 29141660 10.1186/s13059-017-1349-1 PMC5688663

[28] Finotello F , Mayer C , Plattner C , et al. Molecular and pharmacological modulators of the tumor immune contexture revealed by deconvolution of RNA‐seq data. Genome Med. 2019;11(1):34. 31126321 10.1186/s13073-019-0638-6 PMC6534875

[29] Li T , Fan J , Wang B , et al. TIMER: a web server for comprehensive analysis of tumor‐infiltrating immune cells. Cancer Res. 2017;77(21):e108‐e110. 29092952 10.1158/0008-5472.CAN-17-0307 PMC6042652

[30] Racle J , de Jonge K , Baumgaertner P , Speiser DE , Gfeller D . Simultaneous enumeration of cancer and immune cell types from bulk tumor gene expression data. Elife. 2017;6:e26476. 29130882 10.7554/eLife.26476 PMC5718706

[31] Newman AM , Liu CL , Green MR , et al. Robust enumeration of cell subsets from tissue expression profiles. Nat Methods. 2015;12(5):453‐457. 25822800 10.1038/nmeth.3337 PMC4739640

[32] Sturm G , Finotello F , Petitprez F , et al. Comprehensive evaluation of transcriptome‐based cell‐type quantification methods for immuno‐oncology. Bioinformatics. 2019;35(14):i436‐i445. 31510660 10.1093/bioinformatics/btz363 PMC6612828

[33] Cheng Q , Duan W , He S , et al. Multi‐omics data integration analysis of an immune‐related gene signature in LGG patients with epilepsy. Front Cell Dev Biol. 2021;9:686909. 34336837 10.3389/fcell.2021.686909 PMC8322853

[34] Hopkins AM , Kichenadasse G , Karapetis CS , Rowland A , Sorich MJ . Concomitant antibiotic use and survival in urothelial carcinoma treated with atezolizumab. Eur Urol. 2020;78(4):540‐543. 32660748 10.1016/j.eururo.2020.06.061

[35] Yang W , Soares J , Greninger P , et al. Genomics of drug sensitivity in cancer (GDSC): a resource for therapeutic biomarker discovery in cancer cells. Nucleic Acids Res. 2013;41:D955‐D961. 23180760 10.1093/nar/gks1111 PMC3531057

[36] Geeleher P , Cox N , Huang RS . PRRophetic: an R package for prediction of clinical chemotherapeutic response from tumor gene expression levels. Plos One. 2014;9(9):e107468. 25229481 10.1371/journal.pone.0107468 PMC4167990

[37] Reinhold WC , Sunshine M , Liu H , et al. CellMiner: a web‐based suite of genomic and pharmacologic tools to explore transcript and drug patterns in the NCI‐60 cell line set. Cancer Res. 2012;72(14):3499‐3511. 22802077 10.1158/0008-5472.CAN-12-1370 PMC3399763

[38] Johnson DE , Burtness B , Leemans CR , Lui VWY , Bauman JE , Grandis JR . Head and neck squamous cell carcinoma. Nat Rev Dis Primers. 2020;6(1):92. 33243986 10.1038/s41572-020-00224-3 PMC7944998

[39] Denaro N , Russi EG , Merlano MC . Pros and cons of the new edition of TNM classification of head and neck squamous cell carcinoma. Oncology. 2018;95(4):202‐210. 29975952 10.1159/000490415

[40] Allende ML , Amsterdam A , Becker T , Kawakami K , Gaiano N , Hopkins N . Insertional mutagenesis in zebrafish identifies two novel genes, pescadillo and dead eye, essential for embryonic development. Genes Dev. 1996;10(24):3141‐3155. 8985183 10.1101/gad.10.24.3141

[41] Kinoshita Y , Jarell AD , Flaman JM , et al. Pescadillo, a novel cell cycle regulatory protein abnormally expressed in malignant cells. J Biol Chem. 2001;276(9):6656‐6665. 11071894 10.1074/jbc.M008536200

[42] Duffy MJ , Synnott NC , Crown J . Mutant p53 as a target for cancer treatment. Eur J Cancer. 2017;83:258‐265. 28756138 10.1016/j.ejca.2017.06.023

[43] Isayeva T , Li Y , Maswahu D , Brandwein‐Gensler M . Human papillomavirus in non‐oropharyngeal head and neck cancers: a systematic literature review. Head Neck Pathol. 2012;6(Suppl 1):S104‐S120. 22782230 10.1007/s12105-012-0368-1 PMC3394168

[44] Comprehensive genomic characterization of head and neck squamous cell carcinomas. Nature. 2015;517(7536):576‐582. 25631445 10.1038/nature14129 PMC4311405

[45] Gooi Z , Chan JY , Fakhry C . The epidemiology of the human papillomavirus related to oropharyngeal head and neck cancer. Laryngoscope. 2016;126(4):894‐900. 26845348 10.1002/lary.25767

[46] Shield KD , Ferlay J , Jemal A , et al. The global incidence of lip, oral cavity, and pharyngeal cancers by subsite in 2012. CA Cancer J Clin. 2017;67(1):51‐64. 28076666 10.3322/caac.21384

[47] Yang L , Lu P , Yang X , Li K , Chen X , Qu S . Excavating novel diagnostic and prognostic long non‐coding RNAs (lncRNAs) for head and neck squamous cell carcinoma: an integrated bioinformatics analysis of competing endogenous RNAs (ceRNAs) and gene co‐expression networks. Bioengineered. 2021;12(2):12821‐12838. 34898376 10.1080/21655979.2021.2003925 PMC8810019

[48] Esteves LI , Javaroni AC , Nishimoto IN , et al. DNA methylation in the CTCF‐binding site I and the expression pattern of the H19 gene: does positive expression predict poor prognosis in early stage head and neck carcinomas? Mol Carcinog. 2005;44(2):102‐110. 16015666 10.1002/mc.20126

[49] Sun Q , Zhang SY , Zhao JF , Han XG , Wang HB , Sun ML . HIF‐1alpha or HOTTIP/CTCF promotes head and neck squamous cell carcinoma progression and drug resistance by targeting HOXA9. Mol Ther Nucleic Acids. 2020;20:164‐175. 32169804 10.1016/j.omtn.2019.12.045 PMC7068198

[50] Ambatipudi S , Bhosale PG , Heath E , et al. Downregulation of keratin 76 expression during oral carcinogenesis of human, hamster and mouse. Plos One. 2013;8(7):e70688. 23936238 10.1371/journal.pone.0070688 PMC3728316

[51] Sequeira I , Neves JF , Carrero D , et al. Immunomodulatory role of keratin 76 in oral and gastric cancer. Nat Commun. 2018;9(1):3437. 30143634 10.1038/s41467-018-05872-4 PMC6109110

[52] Bloor BK , Tidman N , Leigh IM , et al. Expression of keratin K2e in cutaneous and oral lesions: association with keratinocyte activation, proliferation, and keratinization. Am J Pathol. 2003;162(3):963‐975. 12598329 10.1016/S0002-9440(10)63891-6 PMC1868097

[53] Sulima SO , Hofman I , De Keersmaecker K , Dinman JD . How ribosomes translate cancer. Cancer Discov. 2017;7(10):1069‐1087. 28923911 10.1158/2159-8290.CD-17-0550 PMC5630089

[54] Kampen KR , Sulima SO , Vereecke S , De Keersmaecker K . Hallmarks of ribosomopathies. Nucleic Acids Res. 2020;48(3):1013‐1028. 31350888 10.1093/nar/gkz637 PMC7026650

[55] Damia G , Broggini M . Platinum resistance in ovarian cancer: role of DNA repair. Cancers (Basel). 2019;11(1):119. 30669514 10.3390/cancers11010119 PMC6357127

[56] Eckstein N . Platinum resistance in breast and ovarian cancer cell lines. J Exp Clin Cancer Res. 2011;30(1):91. 21967738 10.1186/1756-9966-30-91 PMC3197542

[57] Kong B , Wang Q , Fung E , Xue K , Tsang BK . P53 is required for cisplatin‐induced processing of the mitochondrial fusion protein L‐Opa1 that is mediated by the mitochondrial metallopeptidase Oma1 in gynecologic cancers. J Biol Chem. 2014;289(39):27134‐27145. 25112877 10.1074/jbc.M114.594812 PMC4175349

[58] de Sa JP , Camara D , Porcacchia AS , et al. The roles of ROS in cancer heterogeneity and therapy. Oxid Med Cell Longev. 2017;2017:2467940. 29123614 10.1155/2017/2467940 PMC5662836

[59] van Gisbergen MW , Voets AM , Starmans MH , et al. How do changes in the mtDNA and mitochondrial dysfunction influence cancer and cancer therapy? Challenges, opportunities and models. Mutat Res Rev Mutat Res. 2015;764:16‐30. 26041263 10.1016/j.mrrev.2015.01.001

[60] Padoan A , Plebani M , Basso D . Inflammation and pancreatic cancer: focus on metabolism, cytokines, and immunity. Int J Mol Sci. 2019;20(3):676. 30764482 10.3390/ijms20030676 PMC6387440

[61] Peltanova B , Raudenska M , Masarik M . Effect of tumor microenvironment on pathogenesis of the head and neck squamous cell carcinoma: a systematic review. Mol Cancer. 2019;18(1):63. 30927923 10.1186/s12943-019-0983-5 PMC6441173

[62] Schoenberg MB , Li X , Li X , et al. The interactions between major immune effector cells and hepatocellular carcinoma: a systematic review. Int Immunopharmacol. 2021;101:108220. 34673334 10.1016/j.intimp.2021.108220

[63] Kumar AT , Knops A , Swendseid B , et al. Prognostic significance of tumor‐associated macrophage content in head and neck squamous cell carcinoma: a meta‐analysis. Front Oncol. 2019;9:656. 31396482 10.3389/fonc.2019.00656 PMC6663973

[64] Yang L , Zhang Y . Tumor‐associated macrophages: from basic research to clinical application. J Hematol Oncol. 2017;10(1):58. 28241846 10.1186/s13045-017-0430-2 PMC5329931

[65] Gao L , Zhang W , Zhong WQ , et al. Tumor associated macrophages induce epithelial to mesenchymal transition via the EGFR/ERK1/2 pathway in head and neck squamous cell carcinoma. Oncol Rep. 2018;40(5):2558‐2572. 30132555 10.3892/or.2018.6657 PMC6151899

[66] Wang SS , Liu W , Ly D , Xu H , Qu L , Zhang L . Tumor‐infiltrating B cells: their role and application in anti‐tumor immunity in lung cancer. Cell Mol Immunol. 2019;16(1):6‐18. 29628498 10.1038/s41423-018-0027-x PMC6318290

[67] Economopoulou P , Agelaki S , Perisanidis C , Giotakis EI , Psyrri A . The promise of immunotherapy in head and neck squamous cell carcinoma. Ann Oncol. 2016;27(9):1675‐1685. 27380958 10.1093/annonc/mdw226

[68] Donskov F . Immunomonitoring and prognostic relevance of neutrophils in clinical trials. Semin Cancer Biol. 2013;23(3):200‐207. 23403174 10.1016/j.semcancer.2013.02.001

[69] Rocken M . Early tumor dissemination, but late metastasis: insights into tumor dormancy. J Clin Invest. 2010;120(6):1800‐1803. 20501952 10.1172/JCI43424 PMC2877965

[70] Kolb HJ , Mittermuller J , Clemm C , et al. Donor leukocyte transfusions for treatment of recurrent chronic myelogenous leukemia in marrow transplant patients. Blood. 1990;76(12):2462‐2465. 2265242

[71] Waldman AD , Fritz JM , Lenardo MJ . A guide to cancer immunotherapy: from T cell basic science to clinical practice. Nat Rev Immunol. 2020;20(11):651‐668. 32433532 10.1038/s41577-020-0306-5 PMC7238960

[72] Jiang P , Gu S , Pan D , et al. Signatures of T cell dysfunction and exclusion predict cancer immunotherapy response. Nat Med. 2018;24(10):1550‐1558. 30127393 10.1038/s41591-018-0136-1 PMC6487502

