# Supplementary figures and images for: PES1 is a biomarker of head and neck squamous cell carcinoma and is associated with the tumor microenvironment
Source: Cancer Med. 2023 Apr 19;12(11):12622–38. doi: 10.1002/cam4.5948 (PMC10278488; doi:10.1002/cam4.5948)

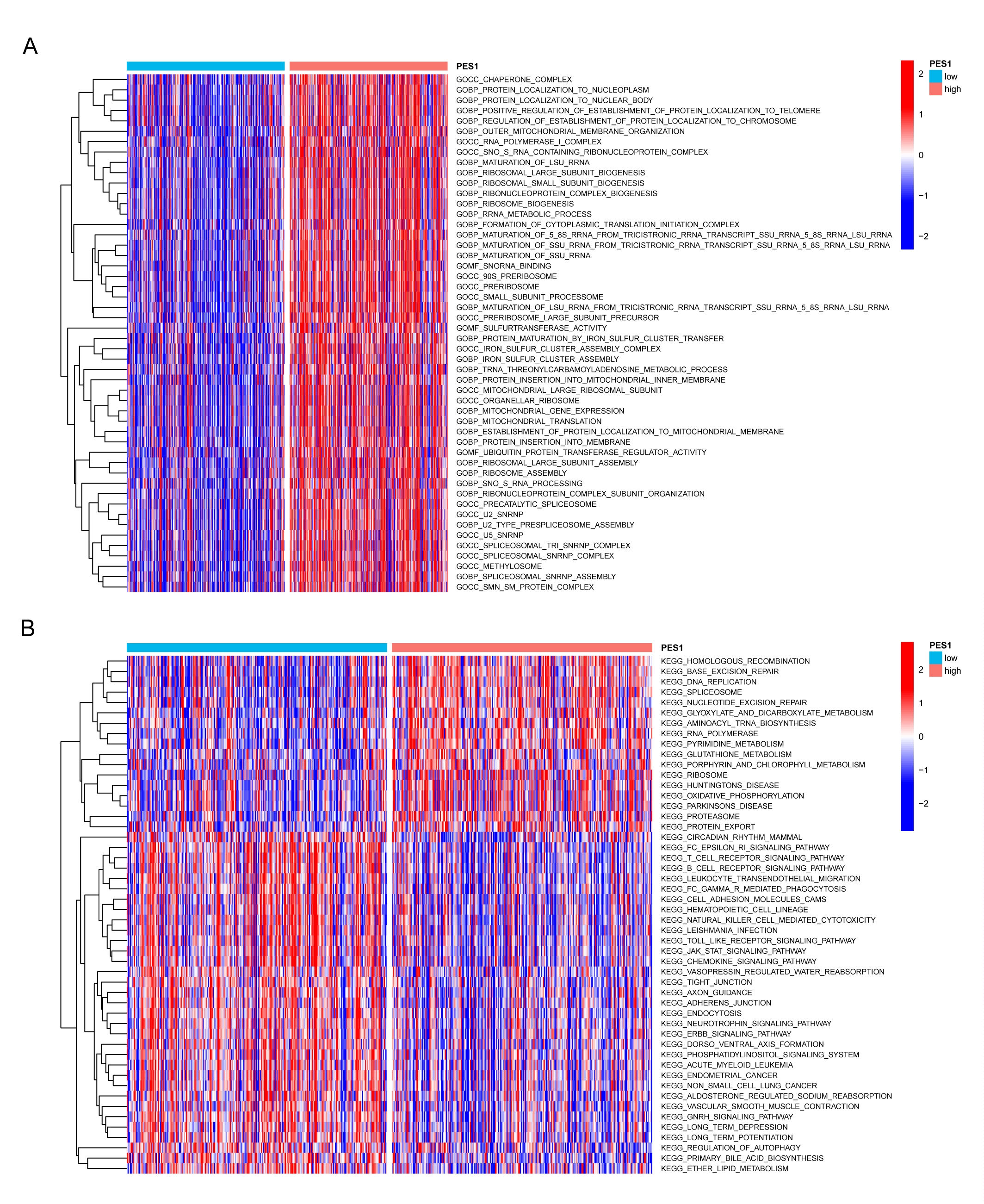

Supplement: Supplementary file 1 — Figure S1. [file CAM4-12-12622-s005.jpeg]

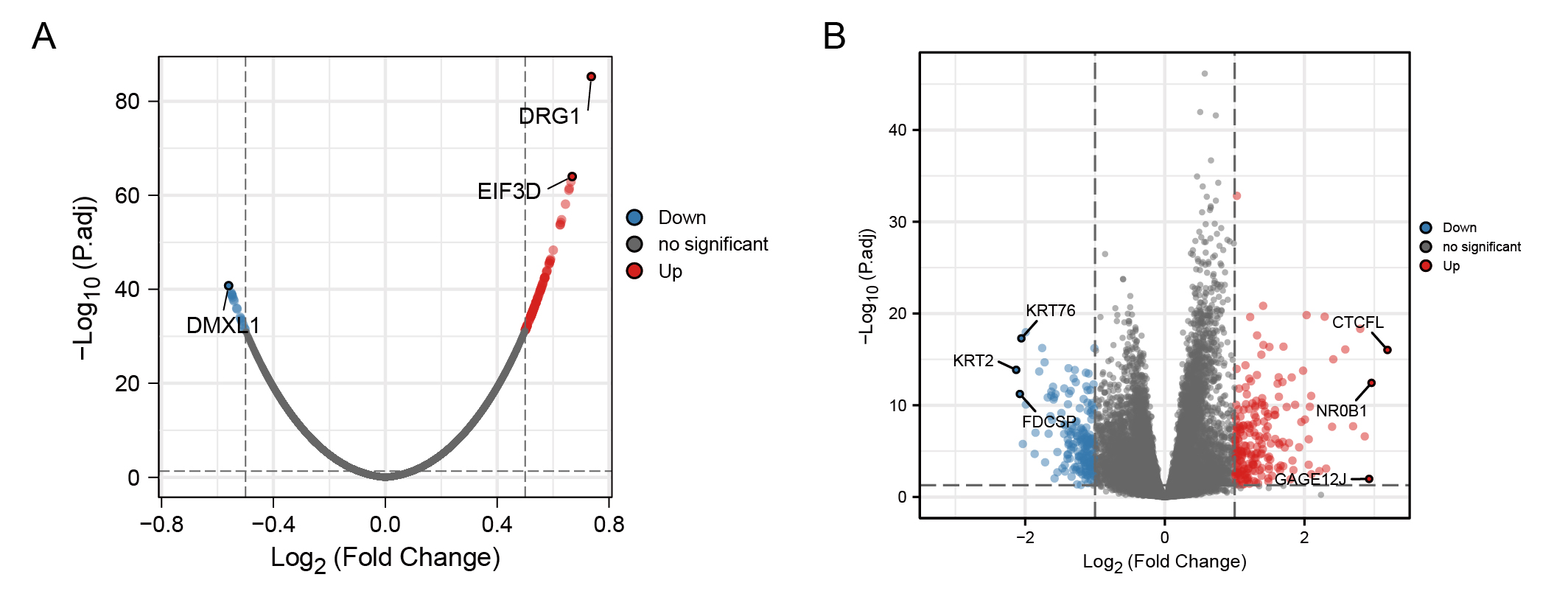

Supplement: Supplementary file 2 — Figure S2. [file CAM4-12-12622-s002.jpg]

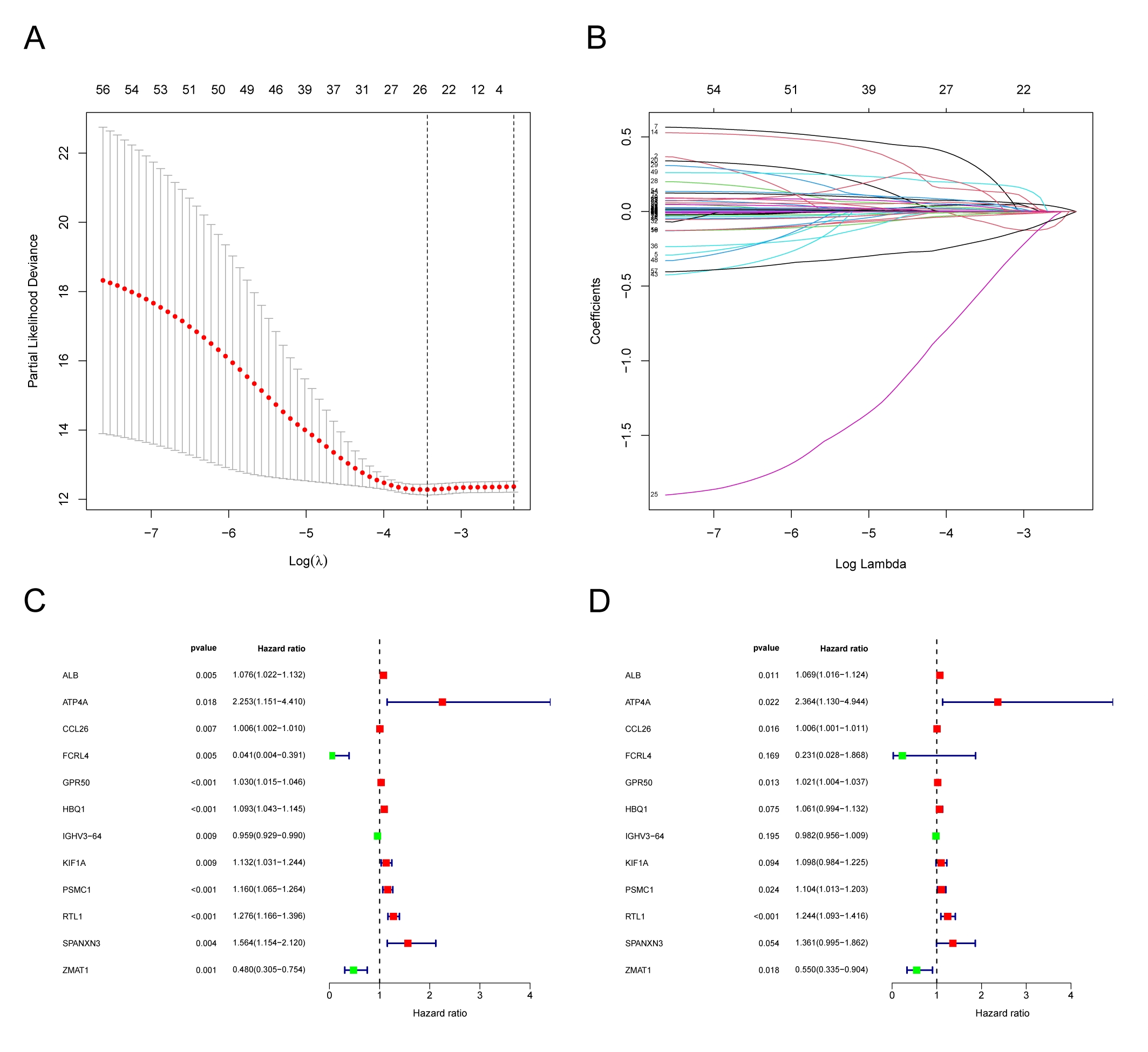

Supplement: Supplementary file 3 — Figure S3. [file CAM4-12-12622-s006.jpeg]
