# Supplementary material for: PES1 is a biomarker of head and neck squamous cell carcinoma and is associated with the tumor microenvironment
Source: Cancer Med. 2023 Apr 19;12(11):12622–38. doi: 10.1002/cam4.5948 (PMC10278488; doi:10.1002/cam4.5948)
Supplement: Supplementary file 4 — Table S1. [file CAM4-12-12622-s001.docx]

**Table S1** A list of primers used in this study

| **Gene** | **Primer** | **Sequence (5' to 3')** |
| --- | --- | --- |
| GAPDH | Forward  Reverse | CAGGAGGCATTGCTGATGAT  GAAGGCTGGGGCTCATTT |
| PES1 | Forward  Reverse | TTAGGCGGCTGTGCATTCTGAAG  GTTGACAATGGGTTCGTGGAGGAG |
